# Rising Ethnic Inequalities in Acute Rheumatic Fever and Rheumatic Heart Disease, New Zealand, 2000–2018

**DOI:** 10.3201/eid2701.191791

**Published:** 2021-01

**Authors:** Julie Bennett, Jane Zhang, William Leung, Susan Jack, Jane Oliver, Rachel Webb, Nigel Wilson, Dianne Sika-Paotonu, Matire Harwood, Michael G. Baker

**Affiliations:** University of Otago, Wellington (J. Bennett, J. Zhang, W. Leung, D. Sika-Paotonu, M.G. Baker);; Southern District Health Board, Dunedin, New Zealand (S. Jack);; University of Melbourne, Melbourne, Victoria, Australia (J. Oliver);; Murdoch Children’s Research Institute, Melbourne (J. Oliver);; Auckland District Health Board, Auckland, New Zealand (R. Webb, N. Wilson);; University of Auckland, Auckland (D. Sika-Paotonu, M. Harwood)

**Keywords:** rheumatic fever, rheumatic heart disease, poverty, children, inequalities, Māori, Pacific Islander, epidemiology, trends, Socioeconomic deprivation, streptococci, bacteria, New Zealand

## Abstract

We describe trends in acute rheumatic fever (ARF), rheumatic heart disease (RHD), and RHD deaths among population groups in New Zealand. We analyzed initial primary ARF and RHD hospitalizations during 2000–2018 and RHD mortality rates during 2000–2016. We found elevated rates of initial ARF hospitalizations for persons of Māori (adjusted rate ratio [aRR] 11.8, 95% CI 10.0–14.0) and Pacific Islander (aRR 23.6, 95% CI 19.9–27.9) ethnicity compared with persons of European/other ethnicity. We also noted higher rates of initial RHD hospitalization for Māori (aRR 3.2, 95% CI 2.9–3.5) and Pacific Islander (aRR 4.6, 95% CI 4.2–5.1) groups and RHD deaths among these groups (Māori aRR 12.3, 95% CI 10.3–14.6, and Pacific Islanders aRR 11.2, 95% CI 9.1–13.8). Rates also were higher in socioeconomically disadvantaged neighborhoods. To curb high rates of ARF and RHD, New Zealand must address increasing social and ethnic inequalities.

## Abstract 2

These conditions disproportionately affect Māori and Pacific Islanders, particularly those living in high socioeconomic deprivation.

Acute rheumatic fever (ARF) is a preventable multisystem inflammatory disease that develops in <3% of persons with untreated group A *Streptococcus* (GAS) pharyngitis (*1*,*2*). Recently, GAS skin infections have been proposed to cause ARF, either directly or in combination with GAS pharyngitis (*3*). The severe sequela of ARF is rheumatic heart disease (RHD) with regurgitation from the mitral valve, aortic valve, or both. RHD is a serious condition that can lead to cardiac failure, stroke, and early death (*4*).

ARF and RHD remain major causes of illness and death (*5*). In 2015, global prevalence of RHD was ≈34 million cases and ≈320,000 RHD-associated deaths occurred (*6*). During the 20th century, improved living conditions resulted in dramatic declines in ARF (*7*). The introduction of antimicrobial drugs in the 1950s and 1960s further reduced the burden of disease and ushered in an effective treatment for GAS pharyngitis (*8*,*9*). Although now rare in high-income countries, ARF and RHD continue to affect populations in economically disadvantaged areas (*10*) and epidemic outbreaks occur in populations that are separated geographically (*11*,*12*).

The incidence of RHD is highest in Oceania, South Asia, and central sub-Saharan Africa (*6*). However, some of the highest reported ARF rates are among indigenous and Pacific Islander populations in Australia and New Zealand (*13*). The incidence rate among indigenous children in Australia in the peak age group, 5–14 years, is 245–351 cases/100,000 population (*14*), but in New Zealand, ARF almost exclusively affects indigenous Māori and Pacific Islander children living in socioeconomically deprived areas of the North Island (*15*,*16*). During 2017–2018, the rate of initial ARF hospitalizations among Māori children 5–14 years of age was 25 cases/100,000 population; among Pacific Islander children, the rate was 81 cases/100,000 population (*17*).

Population-level burden estimates rarely are reported in international literature, partially because of challenges with diagnosing both ARF and RHD and a lack of high-quality surveillance systems for monitoring these conditions. ARF is notifiable to public health authorities in New Zealand, but RHD is not. In addition, historically there has been national undernotification of ARF cases (*18*). Consequently, coded hospitalization data, which is based on the coding system of the International Classification of Diseases (ICD), 9th Revision (ICD-9) and 10th Revision (ICD-10), provides the most comprehensive base for describing ARF and RHD incidence and distribution.

We assessed trends in the incidence of ARF, the frequency of initial hospitalizations for RHD, and RHD mortality rates in New Zealand during 2000–2018. In addition, we assessed the extent to which these conditions are concentrated in specific population groups, based on age, ethnicity, sex, socioeconomic deprivation, and geographic location.

## Methods

### Data Sources

In New Zealand, we can use National Health Index (NHI) numbers (*19*) to identify cases in health data and link information across datasets. We conducted a descriptive epidemiologic study that linked encrypted NHI numbers to ARF and RHD hospital discharge data in New Zealand from 2000 through 2018. To identify cases of initial ARF and recurrent ARF, we used hospital discharge data coded with the ICD in the National Minimum Dataset (*20*), which includes information on all publicly funded hospitalizations in New Zealand.

We defined initial cases as a patient’s first known hospitalization for ARF, which had ICD-10 codes I00, I01, or I02 recorded as their principal diagnosis. We excluded cases in persons who had a previous admission for ARF (ICD-9 codes 390–392) or RHD (ICD-9 codes 393–398) as principal or additional diagnoses since 1988 when the records began. We defined recurrences as all readmissions with ARF as principal diagnosis that occurred >180 days after a previous ARF discharge.

We defined initial RHD cases as a patient’s first hospitalization with a principal diagnosis of RHD (ICD-10 codes I05, I06, I07, I09, or I09) and no previous admission for RHD as principal or additional diagnoses since 1988. We defined RHD death as the underlying cause of death (ICD-10 codes I05, I06, I07, I08, or I09) as recorded in the National Mortality Collection (*21*).

We excluded all non–New Zealand residents from these analyses because they are not part of the usual New Zealand population. We used the New Zealand Deprivation Index (NZDep13) to assess socioeconomic deprivation (*22*). NZDep13 is an area-based measure of socioeconomic deprivation based on 9 variables from the 2013 census. Decile 10 represents areas considered the most socioeconomically deprived, and decile 1 represents areas with the least deprivation. In this article, when we describe the epidemiology of ARF and RHD, we generally are referring to initial ARF or RHD hospitalizations.

### Statistical Analysis

ARF and RHD data were used to calculate the frequencies, rates, rate ratios (RRs), adjusted rate ratios (aRRs), and 95% CIs across selected population groups. To look for time trends, we split the observation into 2 periods: 2000–2009 and 2010–2018 or 2010–2016 for RHD deaths, the timeframe for which mortality data are available. We examined rates of ARF and RHD in relation to characteristics including age, sex, ethnicity, and the district health board (DHB) in which cases occurred. We used DHBs for geographic analysis because they represent the patient’s place of residence. We calculated rates and RRs for ARF for persons <30 years of age because this group accounts for 93.4% of the disease burden. For RHD and RHD mortality, we restricted rates and RRs to persons <70 years of age because the ICD codes are considered to be less specific for RHD in older populations; for example, ICD-10 code I08 includes nonrheumatic heart disease, such as age-related degenerative valvular heart disease that can result in nonrheumatic aortic and mitral valve dysfunction.

We used Poisson regression to calculate RR and 95% CI adjusting for age, sex, ethnicity, and socioeconomic deprivation. We also reported RR by using a defined reference rate from 2000–2009. We used linear regression to examine trends in initial ARF and RHD primary hospitalization rates across most variables. We used the test for trend to evaluate trends over time and considered p<0.05 statistically significant. We performed data analysis by using Excel (Microsoft, https://www.microsoft.com) and SAS 9.4 (SAS Institute, Inc., https://www.sas.com).

Denominator population data were population estimates, calculated from linear interpolation of national census data (*23*). The population of New Zealand at the start of the study was 3.9 million and increased to 4.9 million in 2018. The analysis used the prioritized ethnicity categorizations, which is consistent with national Ministry of Health ethnicity data protocols (*24*). Ethnic groups included in the analysis were Māori, who make up 16.5% of New Zealand’s total population; Pacific Islander (8.1%); Asian (15.1%); and European/other (70.2%). The major Pacific groups in New Zealand are Samoan, Tongan, Cook Island Māori, and Niuean. The major Asian groups are Chinese, Indian, Filipino, and Korean.

### Ethics

The University of Otago Human Research Ethics Committee granted ethical approval for this study (approval no. HD19/033). The Ngāi Tahu Research Consultation Committee also consulted on the study.

## Results

### ARF Incidence Trends and Distribution

During 2000–2018, we noted 2,752 initial hospitalizations with ARF as the principal diagnosis, an average of 145 initial ARF hospitalizations per year. Over the same period, 288 persons were rehospitalized with ARF as their principal diagnosis, an average of 15 recurrent cases per year and 9.5% of the total ARF hospitalizations. Most (47.8%) recurrent cases were among children 10–14 years of age. During 2000–2016, only 3 ARF deaths were recorded, so we did not analyze this outcome further.

**Figure 1:**
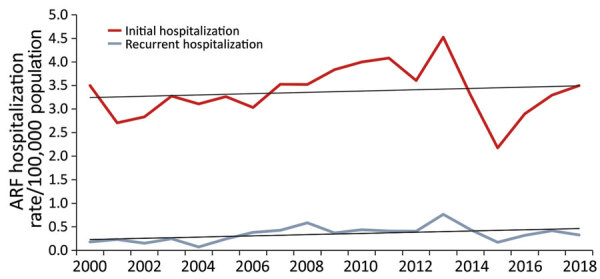
Annual rates of initial and recurrent acute rheumatic fever hospitalizations, New Zealand, 2000–2018. ARF, acute rheumatic fever.

During 2000–2018, annual national initial ARF hospitalization rates ranged from 2.2 to 4.5 cases/100,000 population, an average rate of 3.4 cases/100,000 population (Figure 1). Annual rates of initial ARF hospitalization increased slightly over the study period, but the increase was not statistically significant (p = 0.58). Annual rates of recurrent ARF hospitalizations ranged from 0.1 to 0.8 cases/100,000 population, an average rate of 0.4 cases/100,000 population. Annual rates of recurrent ARF hospitalizations remained stable over time (p = 0.93 by test for trend).

**Figure 2:**
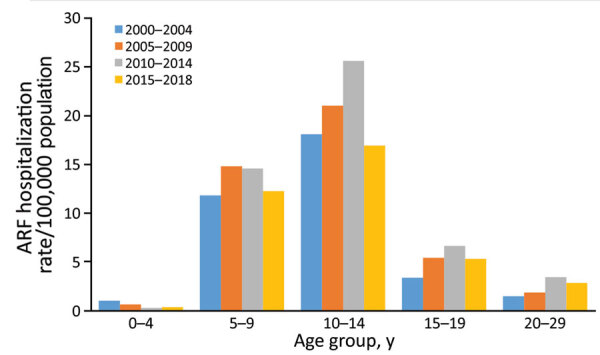
Incidence of initial acute rheumatic fever hospitalizations by age group and time period, New Zealand, 2000–2018. ARF, acute rheumatic fever.

**Figure 3:**
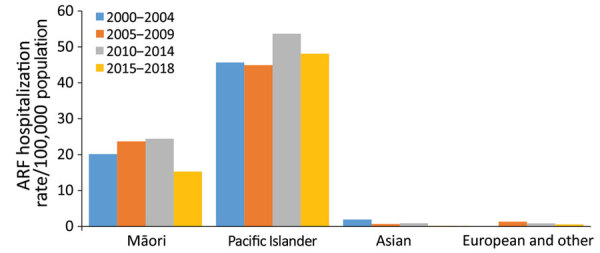
Incidence of initial acute rheumatic fever hospitalizations by major ethnic group and time period among persons <30 years of age, New Zealand, 2000–2018. ARF, acute rheumatic fever.

During 2000–2018, most (93.4%) ARF cases occurred in persons <30 years of age, among whom most (43.0%) children hospitalized with initial ARF were 10–14 years of age, a rate of 20.6 cases/100,000 population (Figure 2). However, 92.6% of initial ARF cases among persons <30 years of age were among Māori or Pacific Islanders; Māori accounted for 48.9% of cases and Pacific Islanders for 43.7%. Pacific Islanders had the highest average initial ARF hospitalization rates among persons <30 years of age, 38.1 cases/100,000 population, and rates among Māori were 16.8 cases/100,000 population (Figure 3).

Initial ARF hospitalization rates peaked at 35.9 cases/100,000 population among Māori children 5–14 years of age and at 79.6 cases/100,000 population for Pacific Islander children. By comparison, rates for European/other ethnicities were 1.6 cases/100,000 population. The net effect of the elevated rates among Māori and Pacific Islander children means that by age 20, the cumulative risk for hospitalized ARF is 1.2% for Pacific Islanders, 0.5% of Māori, and 0.01% for other ethnicities. 

Patients with initial ARF hospitalizations were more likely (aRR 5.2, 95% CI 4.0–6.8) to come from the most socioeconomically deprived areas of the country (NZDep 9–10). The most socioeconomically disadvantaged neighborhoods had greater increases in ARF hospitalizations over time. Independent of socioeconomic deprivation, Pacific Islanders and Māori <30 years of age had markedly higher rates of initial ARF hospitalization than persons in other ethnic groups (Table 1) and rates for Pacific Islanders increased greatly over time (RR 1.3, 95% CI 1.2–1.5).

**Table 1:** Acute rheumatic fever initial hospitalizations and adjusted rate ratios for patients <30 years of age, according to key sociodemographic characteristics, New Zealand, 2000–2018*

Category	No. cases	Crude rate of ARF at initial hospitalization/100,000 population	aRR (95% CI)	RR in 2010–2018 vs. 2000–2009 (95% CI)
Age†				
<5	35	0.62	0.18 (0.13–0.25)	0.42 (0.20–0.88)
5–9	768	13.45	4.10 (3.57–4.70)	1.01 (0.88–1.17)
10–14	1,184	20.55	6.58 (5.77–7.51)	1.08 (0.96–1.21)
15–19	308	5.27	1.81 (1.54–2.13)	1.29 (1.03–1.62)
20–29	276	2.27	Referent	1.86 (1.45–2.38)
Sex‡				
M	1,493	8.60	1.34 (1.24–1.45)	1.09 (0.99–1.21)
F	1,078	6.42	Referent	1.18 (1.05–1.33)
Ethnicity (prioritized)§				
Māori	1257	16.77	11.84 (10.02–13.98)	1.09 (0.98–1.22)
Pacific Islander	1124	38.12	23.57 (19.88–27.94)	1.30 (1.16–1.47)
Asian	23	0.55	0.64 (0.41–0.99)	0.41 (0.18–0.98)
European and other	167	0.86	Referent	0.61 (0.44–0.84)
Socioeconomic deprivation level¶				
1–2	61	1.06	Referent	0.63 (0.38–1.05)
3–4	128	2.16	1.65 (1.21–2.23)	0.85 (0.60–1.20)
5–6	160	2.51	1.60 (1.19–2.25)	0.91 (0.67–1.24)
7–8	405	5.57	2.58 (1.96–3.38)	1.31 (1.08–1.59)
9–10	1,817	20.58	5.21 (4.01–6.75)	1.16 (1.06–1.27)
District health board#				
Northland	206	17.36	7.56 (4.39–13.02)	1.10 (0.84–1.45)
Counties Manukau, South Auckland	909	21.67	7.37 (4.32–12.53)	1.23 (1.08–1.41)
Tairawhiti, Gisborne	63	16.01	5.47(3.06–9.80)	1.45 (0.88–2.39)
South Island, 5 DHBs	67	0.91	Referent	1.21 (0.71–1.88)
Total	2,571	7.53		1.13 (1.04–1.22)

*An additional 181 (6.6% of total) cases occurred among persons >30 years of age during 2000–2018. aRR, adjusted rate ratio; DHB, district health board; RR, rate ratio. †RR adjusted for sex, ethnicity, and socioeconomic deprivation. ‡RR adjusted for age, ethnicity, and socioeconomic deprivation. §RR adjusted for age, sex, and socioeconomic deprivation. ¶RR adjusted for age, sex, and ethnicity. #RR adjusted for age, sex, ethnicity, and socioeconomic deprivation; 3 DHBs highest incidence and 1 DHB with lowest incidence shown. A full list of DHBs is provided in Appendix Table 1).

Rates of initial ARF hospitalizations increased for persons <30 years of age from the 2000–2009 period to the 2010–2018. Increases were statistically significant for persons 15–19 and 20–29 years of age (p<0.05).

Rates of ARF vary throughout regions of New Zealand. Counties Manukau, in South Auckland, had the highest rate for initial ARF hospitalization among persons <30 years of age (21.7 cases/100,000 population), but rates also were high in Northland (17.4 cases/100,000 population). Over the study period, the DHBs of Counties Manukau and the Hutt Valley had statistically significant increases in rates of initial ARF hospitalization (p<0.01) (Appendix Table 1).

### RHD Incidence Trends and Distribution

**Figure 4:**
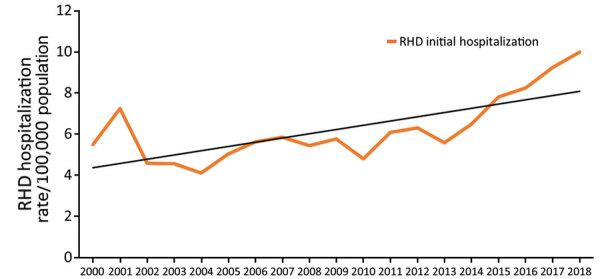
New Zealand annual incidence rates of initial RHD hospitalizations, all ages, 2000–2018. RHD, rheumatic heart disease.

During 2000–2018, a total of 12,094 hospitalizations with a principal diagnosis of RHD were reported, an average of 636 admissions per year. Of these, 5,109 persons were hospitalized with an initial RHD diagnosis, an annual average of 269 persons. During the study period, national initial RHD hospitalization rates ranged from 4.1 to 10.0 cases/100,000 population, an average incidence rate of 6.2 cases/100,000 population (Figure 4). Total rates of RHD hospitalizations as principal diagnosis, including initial and repeat admissions, ranged 11.5 to 17.9 cases/100,000 population, an annual rate of 14.3 cases/100,000 population. The mean age of initial RHD hospitalization was 60 years, with a median age of 67 years.

We analyzed RHD cases according to sociodemographic characteristics for patients aged <70 years (Table 2). We noted that initial RHD primary hospitalization rates increased over time at a statistically significant level (RR 1.09, 95% CI 1.01–1.17; p = 0.03).

**Table 2:** Rheumatic heart disease initial hospitalization rates and adjusted rate ratios for patients <70 years of age according to key sociodemographic characteristics, New Zealand, 2000–2018*

Characteristics	No. cases	Crude rate of RHD at initial hospitalization/100,000 population	aRR (95% CI)	RR in 2010–2018 vs. 2000–2009 (95% CI)
Age, y†				
0–9	154	1.35	Referent	1.53 (1.11–2.11)
10–19	322	2.77	2.21 (1.82–2.68)	1.51 (1.21–1.88)
20–29	164	1.47	1.30 (1.04–1.62)	0.95 (0.69–1.29)
30–39	208	1.87	1.83 (1.49–2.26)	0.81 (0.62–1.07)
40–49	420	3.60	3.84 (3.19–4.62)	1.07 (0.88–1.30)
50–59	678	6.69	7.66 (6.43–9.14)	0.93 (0.80–1.08)
60–69	957	12.87	15.66 (13.18–18.61)	1.13 (1.00–1.29)
Sex‡				
M	1,405	3.74	1.12 (1.04–1.21)	1.14 (1.03–1.26)
F	1,498	4.06	Referent	1.04 (0.93–1.15)
Ethnicity§				
Māori	892	7.30	3.21 (2.93–3.52)	1.24 (1.09–1.42)
Pacific Islander	574	11.60	4.62 (4.16–5.15)	1.28 (1.08–1.51)
Asian	123	1.47	0.71 (0.59–0.86)	0.80 (0.56–1.14)
European and other	1,314	2.68	Referent	0.95 (0.86–1.10)
Socioeconomic deprivation level¶				
1–2	222	1.60	Referent	0.79 (0.60–1.02)
3–4	324	2.34	1.42 (1.20–1.68)	0.85 (0.68–1.05)
5–6	441	3.05	1.76 (1.49–2.06)	1.07 (0.89–1.29)
7–8	642	4.15	2.19 (1.88–2.55)	1.12 (0.56–1.31)
9–10	1,274	7.58	3.10 (2.67–3.60)	1.21 (1.09–1.36)
District health board#				
Northland	150	5.57	1.32 (1.03–1.69)	0.94 (0.68–1.29)
Counties Manukau, South Auckland	478	5.70	1.44 (1.16–1.78)	1.05 (0.88–1.26)
Tairawhiti, Gisborne	90	11.13	2.38 (1.79–3.16)	1.73 (1.12–2.66)
Hutt Valley, Wellington	107	4.33	1.51 (1.15–1.97)	1.38 (0.94–2.03)
Southern, South Island	110	2.14	Referent	1.11 (0.76–1.61)
Total	2,903	3.90		1.09 (1.01–1.17)

*An additional 2,212 (43.2% of total) cases occurred among persons >70 years of age during 2000–2018. aRR, adjusted rate ratio; DHB, district health board; RHD, rheumatic heart disease; RR, rate ratio. †RR adjusted for sex, ethnicity, and socioeconomic deprivation. ‡RR adjusted for age, ethnicity, and socioeconomic deprivation. §RR adjusted for age, sex, and socioeconomic deprivation. ¶RR adjusted for age, sex, and ethnicity. #RR adjusted for age, sex, ethnicity, and socioeconomic deprivation; 4 highest incidence DHBs, and the 1 lowest DHB shown. A full list of DHBs is provided in Appendix Table 1).

We found that risk for RHD is associated with increasing age, Māori and Pacific Islander ethnicity, and socioeconomic deprivation. We also noted a weak association for male over female sex. Rates are markedly higher in some geographic areas (DHBs), but most disappear after adjustment for other sociodemographic factors. Māori and Pacific Islanders made up 50.5% of initial RHD hospitalizations, but RHD rates rose markedly over the observation period for children 0–9 years of age (p = 0.01) and 10–19 years of age (p<0.01) than for any other age groups. 

The initial RHD primary hospitalization rate for Pacific Islanders <70 years of age was 11.6 cases/100,000 population, and this group was 4.6 times more likely to be hospitalized for RHD than persons in the European/other group; Māori were 3.2 times more likely to be hospitalized for RHD (Table 2). Over the study period, rates of RHD rose at statistically significant levels among persons <70 years of age in both Māori (p = 0.01) and Pacific Islander (p = 0.01) populations. Geographically, Counties Manukau DHB had the most cases of RHD and Tairawhiti DHB had the highest rate for initial RHD hospitalizations (Table 2; Appendix Table 1).

Over the study period, persons living in the most socioeconomically deprived areas (NZDep 9–10) had the highest rates of initial hospitalization with a principal diagnosis of RHD, 7.6 cases/100,000 population. The most socioeconomically deprived areas (NZDep 9–10) experienced major increases in initial RHD hospitalizations over the study period (Table 2). Māori and Pacific Islanders living in the most socioeconomically-deprived areas were much more likely to be hospitalized with RHD (Māori aRR 10.58, 95% CI 8.88–12.61; Pacific Islander aRR 13.80, 95% CI 11.48–16.58) than European/other (aRR 3.33, 95% CI 2.76–4.01) or Asian (aRR 1.82, 95% CI 1.25–2.64) populations living in the most socioeconomically deprived areas.

### RHD Mortality Rates and Distribution

During 2000–2016, a total of 2,435 deaths were attributed to RHD, with an average of 143 deaths per year and a rate of 3.4 deaths/100,000 population. The highest rates for RHD coded as the underlying cause of death occurred among persons 60–69 years of age. We noted a 42.6% decline in RHD mortality rates among persons <70 years of age from the 2000–2009 period to the 2010–2016 period.

RHD mortality rates for people <70 years varied according to sociodemographic characteristics (Table 3). The risk for RHD death was most strongly associated with increasing age, Māori and Pacific Islander ethnicity, and socioeconomic deprivation. Pacific Islanders <70 years of age had an average RHD mortality rate of 4.4 deaths/100,000 population and were more likely to die from RHD than persons of European/other ethnicity (aRR 11.2, 95% CI 9.1–13.8); Māori had an RHD mortality rate of 4.3 deaths/100,000 population and were also more likely than European/other to die from RHD (aRR 12.3, 95% CI 10.3–14.6). Among RHD deaths, 73.8% were persons of Māori and Pacific Islander ethnicity.

**Table 3:** Mortality rates of rheumatic heart disease and adjusted rate ratios for people <70 years of age, according to key sociodemographic characteristics, New Zealand, 2000–2016*

Category	No. deaths	Crude rate of RHD deaths/100,000 population	aRR (95% CI)	RR during 2010–2016 vs. 2000–2009 (95% CI)
Age, y†				
<40	126	0.31	Referent	0.66 (0.45–0.96)
40–49	163	1.56	7.27 (5.76–9.18)	0.68 (0.49–0.94)
50–59	249	2.80	15.09 (12.17–18.72)	0.53 (0.41–0.70)
60–69	352	5.48	34.15 (27.80–41.95)	0.56 (0.45–0.69)
Sex‡				
M	394	1.21	0.88 (0.75–0.98)	0.56 (0.45–0.69)
F	496	1.49	Referent	0.61 (0.51–0.73)
Ethnicity§				
Māori	467	4.34	12.27 (10.32–14.58)	0.53 (0.44–0.65)
Pacific Islander	190	4.37	11.16 (9.05–13.76)	0.77 (0.57–1.02)
Asian	20	0.29	0.8 7(0.55–1.38)	0.09 (0.02–0.40)
European and other	213	0.49	Referent	0.63 (0.47–0.84)
Socioeconomic deprivation level¶				
1–2	48	0.39	Referent	0.23 (0.11–0.46)
3–4	67	0.55	1.23 (0.85–1.78)	0.51 (0.30–0.84)
5–6	113	0.88	1.72 (1.23–2.42)	1.01 (0.70–1.46)
7–8	188	1.37	2.17 (1.57–2.99)	0.54 (0.40–0.73)
9–10	474	3.18	3.18 (2.34–4.33)	0.58 (0.48–0.71)
District health board#				
Northland	52	2.18	1.13 (0.76–1.69)	0.48 (0.24–0.94)
Counties Manukau, South Auckland	168	2.28	1.65 (1.21–2.24)	0.75 (0.53–1.05)
Tairawhiti, Gisborne	33	4.56	2.22 (1.42–3.48)	0.35 (0.15–0.83)
South Island, 5 DHBs	81	0.53	Referent	0.50 (0.31–0.82)
Total	890	1.35		0.58 (0.51–0.67)

*An additional 1,545 (63.4% of total) deaths occurred among persons >70 years of age during 2000–2016. aRR, adjusted rate ratio; DHB, district health board; RHD, rheumatic heart disease; RR, rate ratio. †RR adjusted for sex, ethnicity, and socioeconomic deprivation. ‡RR adjusted for age, ethnicity, and socioeconomic deprivation. §RR adjusted for age, sex, and socioeconomic deprivation. ¶RR adjusted for age, sex, and ethnicity. #RR adjusted for age, sex, ethnicity, and socioeconomic deprivation; 3 highest incidence DHBs and the DHB with lowest incidence shown. A full list of DHBs is provided in Appendix Table 1.

Although a decline in RHD mortality rates has occurred across all sociodemographic groups, it has been least apparent among Pacific Islanders. The mean age at RHD death for Māori was 59.2 years, for Pacific Islanders 55.2 years, for Asians 66.0 years, and for European/other ethnicities 80.0 years. RHD death was associated with socioeconomic deprivation; persons living in the most socioeconomically deprived areas were more likely to die from RHD (aRR 3.3, 95% CI 2.3–4.3) than those in the least socioeconomically deprived areas. In addition, Māori living in high deprivation areas (NZDep9–10) were much more likely to die from RHD (aRR 47.52, 95% CI 30.71–73.55), as were Pacific Islanders (aRR 37.46, 95% CI 23.63–59.39). More female (496) than male (394) persons died from RHD over the study period, although this difference was not statistically significant.

### Trends in ARF and RHD by Age and Ethnicity

**Figure 5:**
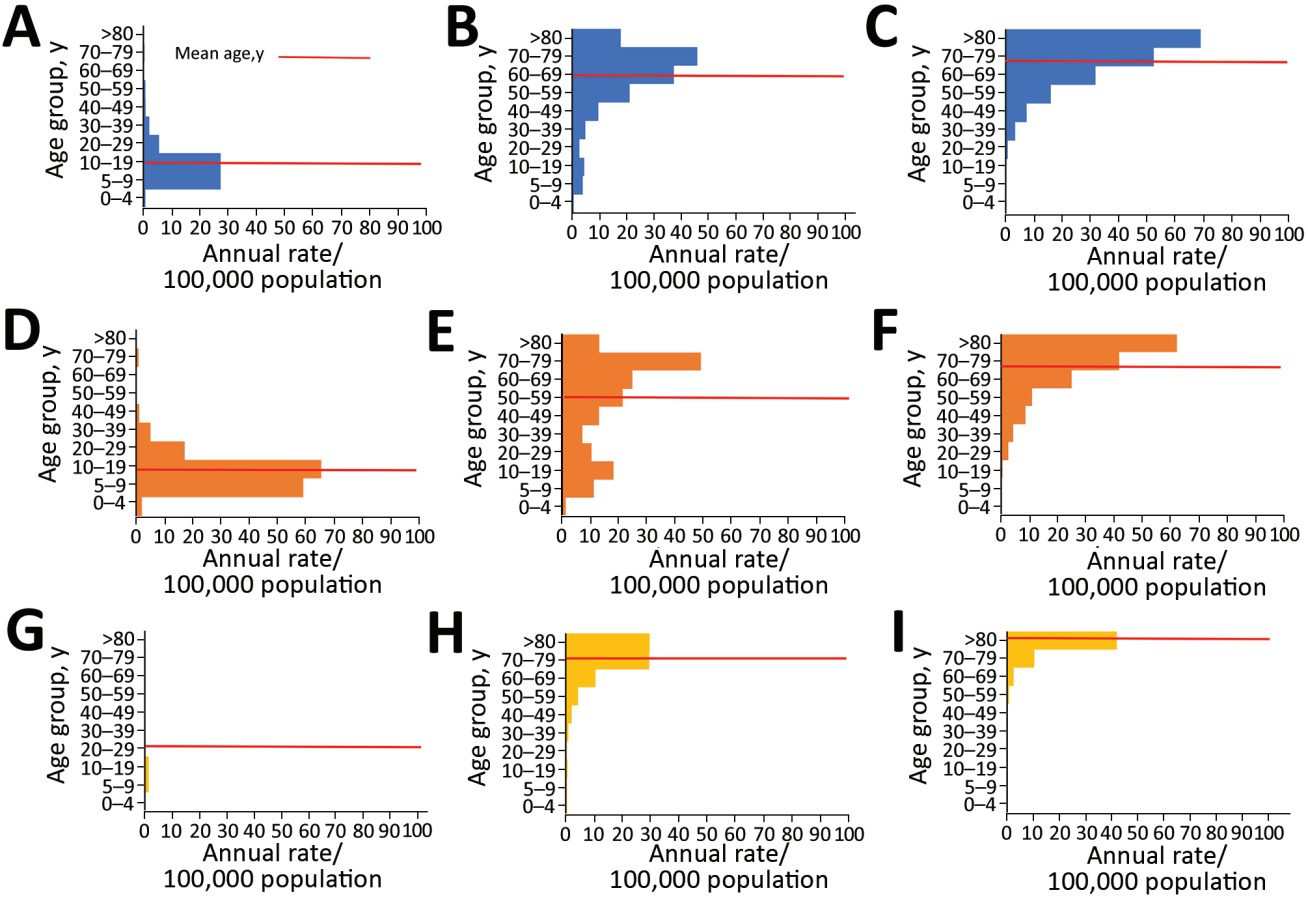
Age distribution of ARF, RHD, and RHD mortality rates across major ethnic groups, New Zealand, 2000–2018. A) ARF incidence among Māori; B) RHD incidence among Māori; C) RHD mortality rates among Māori; D) ARF incidence among Pacific Islanders; E) RHD incidence among Pacific Islanders; F) RHD mortality rates among Pacific Islanders; G) ARF incidence among European or other persons; H) RHD incidence among European or other persons; I) RHD mortality rates among European or other persons. ARF, acute rheumatic fever; RHD, rheumatic heart disease.

We assessed ARF and RHD hospitalizations and RHD deaths by age and ethnic group during 2000–2018 (Figure 5). Māori and Pacific Islander populations suffered the highest rates across all outcomes. In addition, Māori and Pacific Islanders had higher rates of outcomes in younger age groups than European/other or Asian populations.

## Discussion

We provide a comprehensive overview of the epidemiology of ARF, RHD, and death from RHD in New Zealand. Our study builds on previous reports (*15*,*25*–*28*) that examine how the disease burden is shifting and becoming more concentrated in specific population groups.

The overall rate of ARF remained relatively constant during 2000–2018. A marked shift in the distribution demonstrated a major decrease in initial ARF hospitalization rates in persons of European/other ethnicity, but rates have not declined for Māori and have continued to rise for Pacific Islanders. Most (93.4%) initial ARF cases are among persons <30 years of age, and most (92.6%) occur in Māori and Pacific Islanders. Rates of ARF in some population subgroups remain among the highest reported in a high-income country, showing stark ethnic inequalities. For children 5–14 years of age, the rates for Māori (35.9 cases/100,000 population) and Pacific Islanders (79.6 cases/100,000 population) are similar to rates in many low-income countries (*5*,*29**,**30*) and to the high rates previously reported in New Zealand (*25*,*26*). The net effect of these high rates means that, by age 20, the cumulative risk for ARF is 1.2% for Pacific Islanders and 0.5% for Māori, compared with 0.01% for European/other ethnicities. This iniquitous distribution of ARF drives elevated rates of RHD and premature death from RHD across the lifespan for Māori and Pacific Islanders.

Analysis of the sociodemographic characteristics showed ARF almost exclusively affects Māori and Pacific Islander children and young adults. Disaggregating these characteristics shows independent contributions from ethnicity, socioeconomic deprivation, and geographic location (Appendix Table 2). Among persons <30 years of age, Māori had a markedly increased (unadjusted) risk for ARF (RR 19.6, 95% CI 16.7–23.0) as did Pacific Islanders (RR 44.5, 95% CI 37.9–52.4) compared with the European/other group. This elevated risk was reduced after adjustment for socioeconomic deprivation and further reduced after adjustment for geographic location. However, a residual increased risk persisted (RR 9.0, 95% CI 8.2–9.8 for Māori and RR 16.6, 95% CI 14.8–18.6 for Pacific Islanders). A positive finding was that ARF recurrence rates have remained stable over time, with a rate of 0.4 cases/100,000 population. In addition, these cases represent ≈9.5% of total ARF hospitalizations, suggesting successful operation of secondary prevention programs.

RHD hospitalization rates rose greatly over the study period. RHD is concentrated in older age groups and 43.3% of RHD hospitalizations occur among persons >70 years of age. Current trends reflect patterns of ARF that have occurred over the past few decades (cohort effects) and changes in clinical awareness and diagnostic practices. However, Māori and Pacific Islanders again suffer the greatest burden of disease, 50.5% of cases among persons <70 years of age. Ethnic inequalities are less marked for RHD than for ARF, probably reflecting cohort effects from previous decades when ARF inequalities were less marked (*25*). As is the case for ARF, the increased risk for RHD among Māori and Pacific Islanders is associated with deprivation and region in addition to an independent association with ethnicity.

We noted steep declines in RHD mortality rates during 2000–2016. Nonetheless, Māori and Pacific Islanders once more bear the greatest burden of disease, accounting for 73.8% of deaths among persons <70 years of age. As with ARF and RHD hospitalizations, the increased risk for Māori and Pacific Islanders is associated with socioeconomic deprivation and region in addition to an independent association with ethnicity.

A strength of this study is the comprehensive nature of the outcome data used. However, the administrative data used has some limitations, both in identifying and reporting cases of ARF and RHD. Cases can be missed if a person did not seek medical attention, did not have their symptoms recognized as ARF or RHD by a medical professional, or were not hospitalized despite a diagnosis. Consequently, the ARF findings likely underestimate the true incidence of disease. In comparison, RHD hospitalizations might be overestimated due to ICD-10 directives for RHD. Further clinical validation of ICD codes is needed to improve identification of RHD in administrative data in New Zealand and globally. One approach that would greatly help validation would be implementation of a national patient registry, which would enable improved uptake of prophylaxis by patients, better clinical service coordination, and improved healthcare sector performance monitoring. RHD mortality data have similar coding limitations to those seen for RHD hospitalizations. In addition, many RHD deaths are undercounted because they manifest as other circulatory diseases, such as heart failure and strokes (*31*).

Despite these limitations, this study provides a comprehensive overview of the incidence and distribution of ARF, RHD, and RHD deaths in New Zealand. A particularly stark finding is the marked ethnic inequalities in disease burden with ARF disproportionally affecting Māori and Pacific Islander children, and RHD, and RHD deaths disproportionately affecting Māori and Pacific Islander adults, particularly those living in high socioeconomic deprivation. We saw evidence of a cohort effect; new cases of ARF were becoming rare in European/other children, RHD was declining among European/other adults, and RHD deaths were becoming uncommon in European/other persons <70 years of age. However, rates of initial ARF in Māori children remain high and are not decreasing, and rates appear to be rising in Pacific Islander children, condemning these groups to a lifetime living with the effects of RHD. To help curb the continuing high rates of ARF and RHD, New Zealand and other countries must address the large and increasing social and ethnic inequalities.

Appendix Additional information on prevalence of acute rheumatic fever and rheumatic heart disease among ethnic groups, New Zealand.
