# Supplementary material for: Rising Ethnic Inequalities in Acute Rheumatic Fever and Rheumatic Heart Disease, New Zealand, 2000–2018
Source: Emerg Infect Dis. 2021 Jan;27(1):36–46. doi: 10.3201/eid2701.191791 (PMC7774562; doi:10.3201/eid2701.191791)
Supplement: Appendix — Additional information on prevalence of acute rheumatic fever and rheumatic heart disease among ethnic groups, New Zealand. [file 19-1791-Techapp-s1.pdf]

Article DOI: https://doi.org/10.3201/eid2701.191791

Incidence of Acute Rheumatic Fever and Rheumatic Heart Disease among Ethnic Groups, New Zealand, 2000–2018

Appendix

Appendix Table 1. Acute rheumatic fever, initial rheumatic heart disease hospitalization rates, and rheumatic heart disease deaths for District Health Boards, New Zealand, 2000-2016

District health board	No. cases	Average crude rate	Adjusted rate ratio*	Rate ratio (2010–2016 vs. 2000–2009)
Acute rheumatic fever				
Waitemata (North/East Auckland)	197	4.7	3.3 (2.5–4.4)	1.1 (0.8–1.4)
Auckland	281	7.5	4.0 (3.0–5.2)	1.0 (0.8–1.3)
Waikato	226	7.6	3.4 (2.7–4.7)	1.1 (0.9–1.5)
Lakes (Rotorua/Taupo)	99	11.9	3.7 (2.7–5.1)	1.2 (0.8–1.8)
Bay of Plenty	153	10.0	4.1 (3.0–5.5)	0.8 (0.6–1.1)
Hawkes Bay	95	8.0	2.8 (2.1–3.9)	0.8 (0.5–1.1)
Taranaki	20	2.4	1.6 (1.0–2.6)	0.9 (0.4–2.0)
Midcentral (Manawatu)	37	2.8	1.4 (1.0–2.1)	1.1 (0.6–2.1)
Whanagnui	27	5.6	2.1 (1.4–3.3)	1.5 (0.7–3.1)
Capital and Coast (Wellington)	112	4.9	3.1 (2.3–4.2)	1.2 (0.8–1.7)
Wairarapa	7	2.5	1.4 (0.6–3.0)	0.4 (0.1–2.2)
Rheumatic heart disease				
Waitemata (North/East Auckland)	247	2.7	1.2 (1.0–1.5)	1.1 (0.8–1.3)
Auckland	291	3.7	1.4 (1.2–1.8)	1.0 (0.8–1.2)
Waikato	350	5.6	1.8 (1.5–2.3)	1.4 (1.1–1.7)
Lakes (Rotorua/Taupo)	103	5.8	1.5 (1.2–2.0)	1.1 (0.7–1.6)
Bay of Plenty	128	3.7	1.1 (0.8–1.4)	1.3 (0.9–1.9)
Hawkes Bay	125	4.7	1.4 (1.1–1.8)	1.1 (0.8–1.6)
Taranaki	112	6.0	2.2 (1.7–2.9)	0.2 (0.1–0.4)
Midcentral (Manawatu)	138	4.9	1.6 (1.3–2.1)	1.2 (0.8–1.6)
Whanagnui	46	4.3	1.2 (0.8–1.7)	1.2 (0.7–2.1)
Capital and Coast (Wellington)	170	3.4	1.5 (1.1–1.9)	1.2 (0.9–1.6)
Wairarapa	24	3.5	1.2 (0.8–1.8)	2.3 (0.9–5.5)
Nelson	63	2.7	1.1 (0.8–1.6)	2.6 (1.5–4.6)
West Coast (South Island)	13	2.4	0.9 (0.5–1.6)	1.5 (0.5–4.7)
Canterbury	143	1.7	0.8 (0.6–1.1)	0.9 (0.6–1.2)
South Canterbury	15	1.6	0.7 (0.4–1.2)	0.8 (0.3–2.3)
Rheumatic heart disease deaths				
Waitemata (North/East Auckland)	56	0.7	1.1 (0.7–1.6)	1.0 (0.6–1.8)
Auckland	77	1.1	1.4 (1.0–2.0)	0.7 (0.4–1.1)
Waikato	113	2.0	1.9 (1.4–2.6)	0.5 (0.3–0.8)
Lakes (Rotorua/Taupo)	49	3.0	1.7 (1.1–2.6)	0.5 (0.2–0.9)
Bay of Plenty	52	1.7	1.3 (0.9–1.9)	0.3 (0.2–0.7)
Hawkes Bay	48	2.0	1.5 (1.0–2.2)	0.8 (0.4–1.5)
Taranaki	23	1.4	1.7 (1.1–2.8)	0.4 (0.2–1.1)
Midcentral (Manawatu)	43	1.7	1.7 (1.1–2.6)	0.4 (0.2–0.9)
Whanganui	12	1.3	0.8 (0.4–1.7)	0.9 (0.2–3.4)
Capital and Coast (Wellington)	48	1.1	1.5 (1.0–2.2)	0.4 (0.2–0.9)
Hutt Valley (North Wellington)	28	1.3	1.2 (0.7–1.9)	0.5 (0.2–1.2)
Wairarapa	7	1.2	1.5 (0.7–3.2)	0.4 (0.1–2.3)

*Ratio rate adjusted for age, sex, ethnicity, and socioeconomic deprivation.

Appendix Table 2. Rates of hospitalization for acute rheumatic fever and rheumatic heart disease during 2000–2018, and rheumatic heart disease deaths during 2000–2016 across ethnic groups showing effect of different levels of adjustment for sociodemographic characteristics, New Zealand*

Ethnicity	No.	Rate	Crude rate ratio	aRR†	aRR‡	aRR§
Initial ARF <30 years of age						
Māori	1,257	16.8	19.6 (16.7–23.0)	18.7 (16.1–38.1)	11.8 (10.0–14.0)	9.0 (8.2–9.8)
Pacific Islander	1,124	38.1	44.5 (37.9–52.4)	43.9 (37.3–51.7)	23.6 (19.9–27.9)	16.6 (14.8–18.6)
Asian	23	0.6	0.6 (0.4–1.0)	0.7 (0.5–1.2)	0.6 (0.4–1.0)	0.7 (0.5–0.9)
European and other	167	0.9	Referent	Referent	Referent	Referent
Total	2,571	7.5				
Initial RHD <70 years of age						
Māori	892	7.3	2.7 (2.5–3.0)	4.2 (3.6–4.6)	3.2 (2.9–3.5)	3.0 (2.8–3.3)
Pacific Islander	574	11.6	4.3 (3.9–4.8)	6.6 (6.0–7.3)	4.6 (4.2–5.1)	4.5 (4.0–5.1)
Asian	123	1.5	0.6 (0.5–0.7)	0.8 (0.6–0.9)	0.7 (0.6–0.9)	0.7 (0.6–0.8)
European and other	1,314	2.7	Referent	Referent	Referent	Referent
Total	2,903	3.9				
RHD death <70 years of age						
Māori	467	4.3	8.9 (7.6–10.5)	16.6 (14.0–19.7)	12.3 (10.3–14.7)	11.2 (9.2–13.6)
Pacific Islander	190	4.4	9.0 (7.4–10.9)	14.1 (11.3–17.4)	9.4 (7.5–11.9)	7.9 (5.9–10.5)
Asian	20	0.3	0.6 (0.4–0.9)	0.7 (0.5–1.3)	0.7 (0.4–1.2)	0.7 (0.4–1.3)
European and other	213	0.5	Referent	Referent	Referent	Referent
Total	890	1.4				

*ARF, acute rheumatic fever; aRR, adjusted rate ratio; RHD, rheumatic heart disease.
†RR adjusted for age.
‡RR adjusted for age, sex, and socioeconomic deprivation.
§RR adjusted for age, sex, socioeconomic deprivation, and district health board.

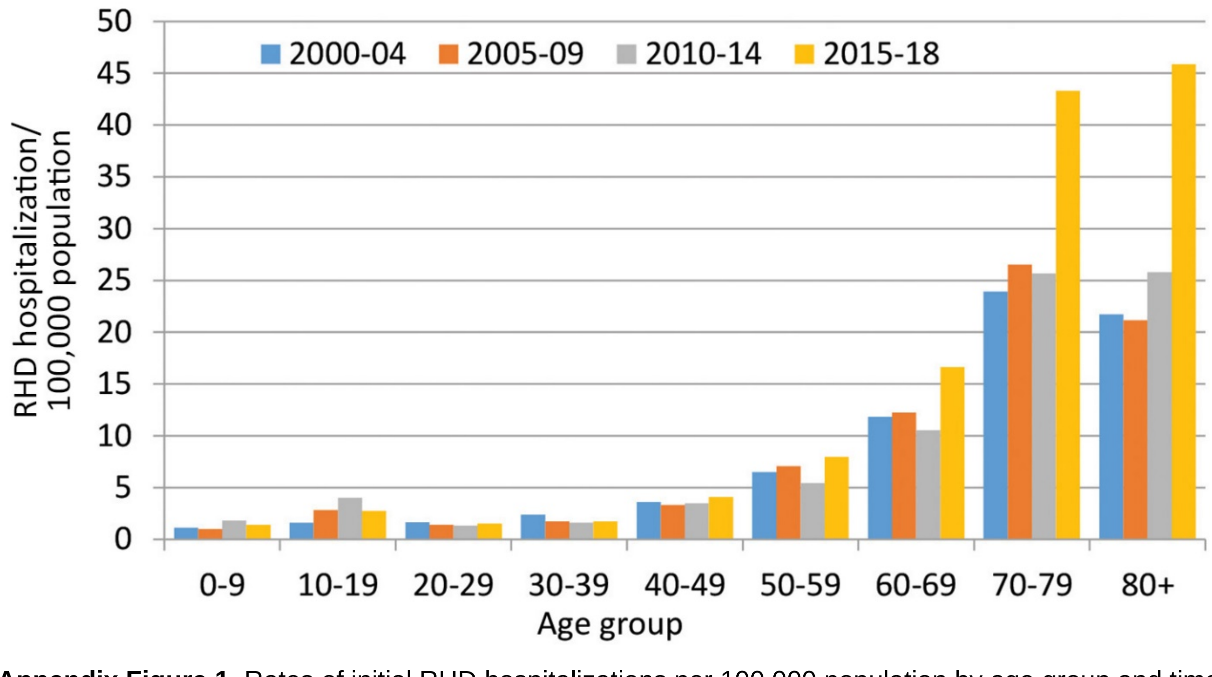

Appendix Figure 1. Rates of initial RHD hospitalizations per 100,000 population by age group and time, New Zealand, 2000–2018. RHD, rheumatic heart disease.

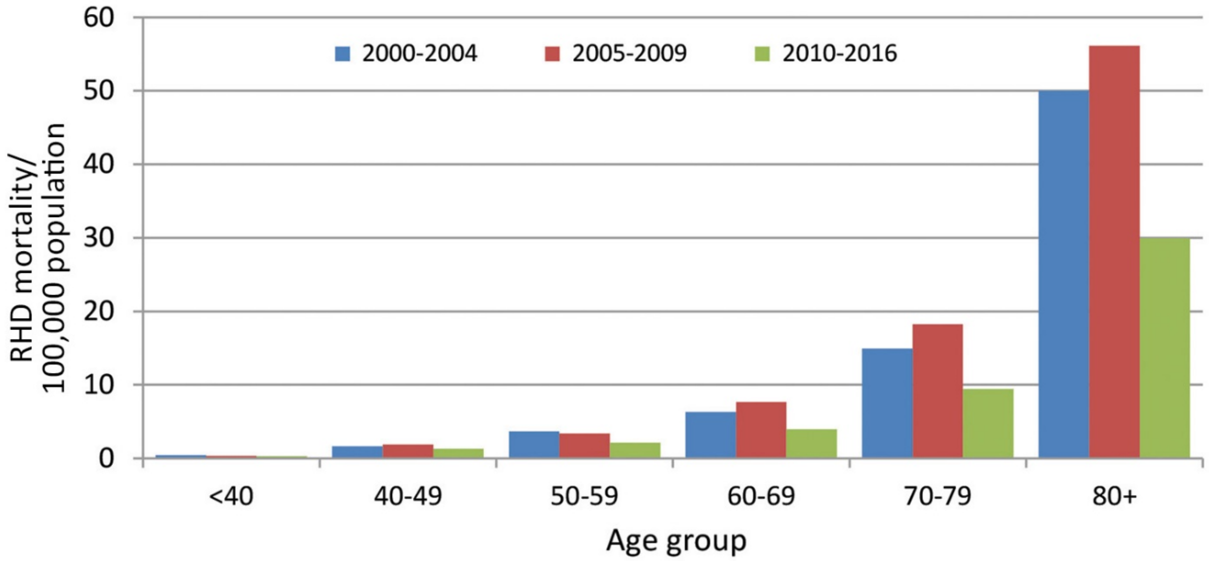

Appendix Figure 2. RHD mortality/100,000 population by age group and time, New Zealand, 2000–2016. RHD, rheumatic heart disease.
